# Influence of Oral Calcium Intake During Pregnancy on Blood and Urine Calcium Levels of Newborns

**DOI:** 10.5812/ircmj.3632

**Published:** 2013-07-05

**Authors:** Sedigheh Ebrahimi, Soheil Ashkani Esfahani

**Affiliations:** 1 Department of Ethics, Faculty of Medicine, Shiraz University of Medical Sciences, Shiraz, Iran; 2 Student Research Committee, Shiraz university of Medical Sciences, Shiraz, Iran

**Keywords:** Pregnancy, Infant, Newborn, Hypercalciuria, Hypocalcaemia

*Dear Editor,*

Pregnancy is a time of increased need for supplementations like calcium. This theory was already supported that maternal calcium supplements during pregnancy was found to bring about a remarkable increase in the density of all the bones in the neonates; this suggests the fact that increased calcium had been actively transferred to the fetus via the placenta (1). In recent years, use of calcium supplements during pregnancy, especially in the last trimester have been recommended to prevent disadvantages of calcium loss by mother and also providing sufficient calcium supplement for the fetus (2, 3). There is a theoretical concern that the excessive consumption of calcium supplements may induce hypocalcaemia in the fetus and neonate, that can be a cause of hypercalciuria, which may be followed by urinary calculi and nephrocalcinosis (4). Recently, we experienced noticeable incidence of hypercalciuria, nephrolithiasis and nephrocalcinosis in healthy newborns. In the present study we investigated the influence of mother’s calcium supplementation in generating hypocalcaemia and hypercalciuria in neonates that were born in hospitals of Yasuj University of medical sciences (south-west of Iran).

Between September 2010 and June 2011, seventy pregnant women and their healthy full term newborns were recruited. Mothers were randomly divided into two groups of thirty-five cases while those with any systemic diseases such as diabetes, hypertension, renal or liver diseases, and those who had used medication that would affect calcium metabolism were excluded. The case group received 1000 mg calcium carbonate daily in the last month of pregnancy; the control group with similar conditions consumed their usual diet. At the time of delivery, cord blood was collected to check for total calcium. First urine, in healthy newborns was checked for Ca/Cr ratio by the cresolphthalein complexone spectrophotometric method and kinetic jaffe reaction, respectively (5). Automated analyzer (Hitachi™ 704) was used, and then urinary Ca/Cr (mg/mg) ratio was calculated. Quantitative values between the two groups were determined by Student t tests by SPSS^®^ software (version 17.0, IBM^®^, USA). Results were reported as Mean ± standard deviation (SD). Values of P ≤ 0.05 were considered as statistically significant. Results demonstrated that average serum calcium in case group was 9.92 ± 1.07 mg/dl (normal level: 8.5-12 mg/dl) and in the control group was 10.05 ± 0.81 mg/dl (normal level: 8.5 - 12.5 mg/dl) which had insignificant difference (P = 0.565) (Table 1).

**Table 1.** Average Serum Calcium (mg/dl; Mean ± SD) of Newborns With and Without Maternal Calcium Supplementation

Groups (n = 35)	Average Serum Calcium	Infants With Normal Calcium Range	Infants with Calcium Range More Than Normal
**Case**	9.92 ± 1.07^a^	74.1%	25.9%
**Control**	10.05 ± 0.81	79.9%	20.1%

^a^ P = 0.565 case group vs. control group

Calcium to creatinin ratio in case group was at a range of 0.05 to 0.21 with average of 0.11 ± 0.05. This ratio in control group was from 0.05 to 0.19 with average of 0.10 ± 0.036, respectively (P = 0.35) (Table 2).

**Table 2.** Urinary Calcium to Creatinin Ratio (mg/mg; Mean ± SD) in Newborns With and Without Maternal Calcium Supplementation

Group (n = 35)	Urine Ca/Cr	Infants With Normal Ca/Cr ratio	Infants With Ca/Cr ratio More Than Normal
**Case**	0.11 ± 0.05^a^	97.1%	2.9%
**Control**	0.10 ± 0.036	100%	0%

^a^ P = 0.352 case group vs. control group

Calculated relative risk test of increasing serum calcium was 1.07 with confidence interval of 95%: (0.35, 2.12), and for calcium to creatinin ratio of serum was 1.03 with confidence interval of 95%: (0.21, 2.67). Maternal and fetal skeletal benefits of calcium supplementation, many other advantages have been linked to adequate calcium intake, such as reduced risk of pregnancy-induced hypertension and relative risk of preeclampsia, a decrease in maternal circulating lead, higher newborn weight and bone mineralization (6-9). Literature and clinical experiences declare the fact that investigations are still needed to prove the safety and to find out the possible disadvantages of maternal calcium supplementation in the neonates. Likely, as results of this study revealed, calcium supplementation had no significant impact in increasing the risk of hypercalciuria and hypocalcaemia occurrence in neonates. The present findings imply that percentage of neonates of case-group with normal calcium level was almost similar to control group and no one in both groups had low calcium level. Moreover, high calcium level proportion in both groups was not noticeably different. Percentage of Ca/Cr ratio within and higher than normal range in case group was not remarkably different from that of control group respectively; thus, this was not a causative factor of the recently observed hypercalciuria in our experiences.
